# Aging, Nutritional Status and Health

**DOI:** 10.3390/healthcare3030648

**Published:** 2015-07-30

**Authors:** Wilma Leslie, Catherine Hankey

**Affiliations:** Human Nutrition, School of Medicine, College of Medical, Veterinary & Life Sciences, University of Glasgow, Glasgow G31 2ER, UK; E-Mail: wilma.leslie@glasgow.ac.uk

**Keywords:** under nutrition, older adults, obesity, nutritional screening and intervention

## Abstract

The older population is increasing worldwide and in many countries older people will outnumber younger people in the near future. This projected growth in the older population has the potential to place significant burdens on healthcare and support services. Meeting the diet and nutrition needs of older people is therefore crucial for the maintenance of health, functional independence and quality of life. While many older adults remain healthy and eat well those in poorer health may experience difficulties in meeting their nutritional needs. Malnutrition, encompassing both under and over nutrition increases health risks in the older population. More recently the increase in obesity, and in turn the incidence of chronic disease in older adults, now justifies weight management interventions in obese older adults. This growing population group is becoming increasingly diverse in their nutritional requirements. Micro-nutrient status may fluctuate and shortfalls in vitamin D, iron and a number of other nutrients are relatively common and can impact on well-being and quality of life. Aging presents a number of challenges for the maintenance of good nutritional health in older adults.

## 1. Introduction

Improvements in public health and medical care are well acknowledged factors in the large improvements in infant and childhood mortality observed in the first half of the 20th century. Increased longevity in adults is also now increasingly common in the developed world. These demographic changes have resulted in increasing numbers and hence proportions of the adult population aged over the age of 60. The time when older people will outnumber younger people is rapidly approaching, it is estimated that by the year 2025 the number of people worldwide aged 60 and over will exceed 1.2 billion [1]. This projected growth in the older population will create significant additional demands on healthcare and support services [2].

Diet and lifestyle, coupled with maintenance of a healthy body weight are important in the maintenance of health for all age groups but are crucial for healthy aging. Maintaining a good nutritional status has significant implications for health and wellbeing, delaying and reducing the risk of developing disease, maintaining functional independence and thus promoting continued independent living [3].

## 2. Nutritional Needs and Changes with Advancing Years

**Figure 1:**
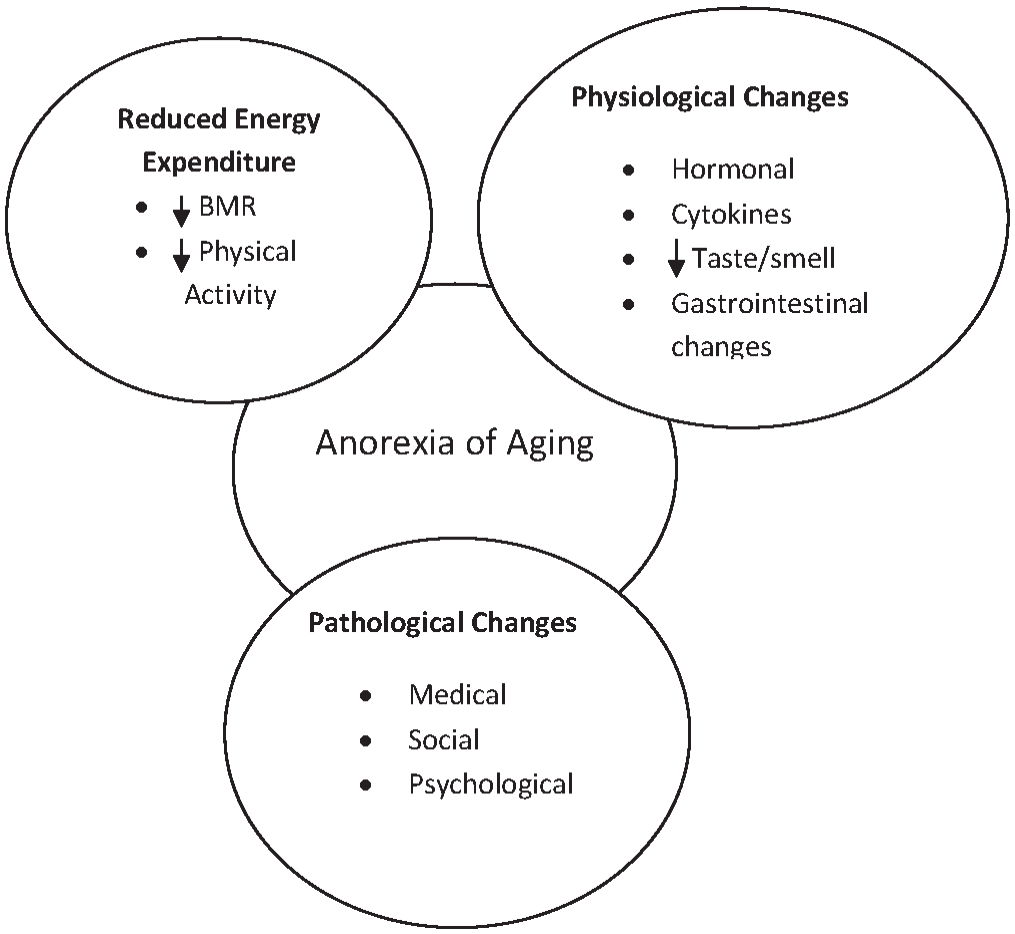
Factors which challenge nutritional status in older adults. Adapted from [4].

Aging is accompanied by many changes that can make it more difficult for nutritional needs to be met. These changes have been categorised into broad categories of physical/physiological and psychosocial (Figure 1) [4].

## 3. Social Factors

For some, good nutrition may become less important with age. Factors such as bereavement, social isolation can influence dietary practices. Cooking a proper meal for one takes time and may feel burdensome and as a consequence meals may become limited to snacks. Illness and disability may also affect the ability to shop for, and prepare food [5].

## 4. Chronic Illness

Aging is accompanied by an increased likelihood of suffering from one, or more, chronic diseases such as respiratory disease, arthritis, stroke, depression and dementia. These conditions may affect appetite, functional ability or ability to swallow, all leading to altered food intake and impairment of nutritional status.

Medications used in the treatment of chronic illness can also have a detrimental effect on nutritional status through loss of appetite, nausea, diarrhoea, reduced gastrointestinal motility and dry mouth [6,7].

## 5. Physiological Changes

Taste and smell diminish with age and poor dentition may limit food choice to soft foods. Dry mouth (xerostomia) is common, making swallowing difficult with subsequent avoidance of foods. Malabsorption of essential nutrient may result as a result of gastrointestinal changes such as atrophic gastritis. Gastric emptying slows with aging with a potential detrimental effect on appetite. All of these factors, independently or collectively, can lead to a reduction in food intake [5].

As we age body composition changes—fat mass increases and lean body mass (muscle) decreases (sarcopenia). Loss of muscle mass begins at around age 50 but becomes more accelerated after the age of 60 years of age, and fat mass continues to increase until around the age of 75 years [8]. Loss of muscle mass leads to a reduction in basal metabolic rate by approximately 15% between the age of 30 and 80, and this results in a subsequent reduction in energy requirements, of around 150kcal per day after the age of 75 [9], Table 1.

**Table 1:** Estimated Average Requirements (EARs) for Energy. Adapted from [9].

EARs MJ/d (kcal/d)		
Age (Years)	Males	Females
19–50	10.60 (2550)	8.10 (1940)
51–59	10.60 (2550)	8.00 (1900)
60–64	9.93 (2380)	7.99 (1900)
65–74	9.71 (2330)	7.96 (1900)
75+	8.77 (2100)	7.61 (1810)

Reductions in energy requirements impact on the quantities or volumes of food consumed, people tend to naturally eat less and this in tandem with the physiological changes described, can lead to shortfalls in micronutrients intakes.

A study of older adults living independently in Eire found shortfalls in intakes of vitamin C and calcium plus, vitamin D, folate, zinc and magnesium. Lowered intakes were particularly evident in those aged 75 years and over [10]. Interestingly the shortfall in micronutrient intakes was accompanied by a high prevalence of overweight and obesity (70%), suggesting energy dense but micro-nutrient poor food intakes in this group. The issue low micronutrient intakes is highlighted by the example of vitamin D. It is notoriously challenging to provide sufficient from food sources and most of our requirements are met through the effect of ultra violet light on the skin. UK dietary survey data [11] has shown vitamin D intakes, from food sources, for men and women aged 65 and over, to be deficient, at only 33% of the Reference Nutrient Intake value. In France, a study which aimed to characterise a frail population of free living adults aged over 65 years [12] found almost everyone (>95% of participants) had a clinical vitamin D deficiency.

Vitamin D is essential for the maintenance of bone health and muscle strength and deficiency in older adults may impact on functional capacity and increase the risk of falls. Vitamin D supplementation of 10 mcg/day [13] is recommended for older adults, especially those who spend little time outside. However a meta-analysis has reported that supplementation of 700–1000 IU (17.5–25 ucg) vitamin D daily reduced risk of falling by 19%, whilst a lower dose of 10 mcg/400 IU was unlikely to reduce the risk of falling among older individuals [14]. Nordic nutritional guidelines advocate vitamin D supplementation for older individuals of 20 mcg daily [15], which may be sufficient to impact on muscle strength [16]. Many diverse populations have reflected the tendency for older adults to have low micronutrient status [17].

## 6. Health Consequences of under and over Nutrition in Older Adults

Older people are vulnerable to malnutrition which is associated with an increased risk of morbidity and mortality [18]. Increased falls, vulnerability to infection, loss of energy and mobility, poor wound healing and confusion are reported consequences of undernutrition [19]. In the UK the health and social care costs associated with undernutrition are reported at around £13 billion per annum [20]. Malnutrition is common in all types of institutional care settings, however much of the malnutrition present on admission to institutions is thought likely to originate in the community among free living older adults. In the UK the prevalence of malnutrition in patients admitted to hospital from home is reported to be 23% [20]. A small US study which aimed to improve the recognition of undernutrition in community dwelling older adults identified 4% with malnutrition and a further 56% at high risk [21]. Social deprivation is one of many factors likely to contribute to this. Those with low incomes are known to have a poorer diet than the more affluent [22] and patients at risk of malnutrition on admission to hospital were found more likely to have come from areas of deprivation [23]. In Scotland around 16% of older people (>65 years) currently live in poverty [24].

Whilst undernutrition may be considered a greater risk to health in older people, obesity also increases morbidity and mortality from diabetes, hypertension and cardiovascular disease. The prevalence of overweight and obesity continues to rise amongst the population as a whole, and current evidence indicates that the prevalence in those aged 65+ is increasing. Scottish Health Survey data has shown that between 1998 and 2008, BMI continued to rise between the age of 60 and 70, especially in women [25]. European and USA data show similar trends [26]. This is in marked contrast to earlier decades when obesity was less common and prevalence increased with age, peaking around age 60 and then declining [25].

## 7. Approaches to Challenge Sub-Optimal Nutritional Status

Recognition of deteriorating or poor nutritional status is key to reversing any effect. Many screening tools have been validated for use in older adults and are available [27]. In the UK the most widely used screening tool is the Malnutrition Universal Screening Tool (MUST), a five step screening tool that includes guidelines for the formulation of a care plan. Across Europe the Mini Nutritional Assessment tool (MNA-SF) is more widely used and was developed specifically for use in older adult. The MNA-SF detected undernutrition in frail elderly in greater numbers than MUST [27]. These differences highlight the disparity between screening tools, and may suggest that MUST is less useful in this group. Both tools collect slightly different information; MUST develops a risk of malnutrition score based upon current body mass index (BMI), known weight loss and the presence of acute disease/no nutritional intake for 5 days. MNA-SF, includes similar questions to the MUST with additional questions on neuropsychological functional status, physical mobility and food intake.

However nutritional screening policies and practice vary between and within health care settings and, despite the availability of screening tools, nutritional screening is often not undertaken and malnutrition continues to be under-recognised and under-treated [21,28]. Screening alone will accrue no benefits for people if action on findings is not taken. This was highlighted in the study by Wadas-Enright and King [21] who found that despite screening which identified subjects as malnourished or at high risk; no referrals for nutritional intervention were made.

Meeting the diet and nutrition needs of older people is crucial for the maintenance of health, functional independence and quality of life. For some living at home an approach as simple as the provision of meals has been shown to be sufficient to improve nutritional status. Improvements in dietary patterns and nutrient intakes were observed in those in receipt of home delivered meals in comparison to those not receiving meals [29]. A study which looked at the effect of two models of “Meals on Wheels” on the nutritional status of housebound older adults found improvements in both groups over a 6 month period. The greatest improvements were seen in the group who received the enhanced programme of meals which included three meals and two snacks per day [30]. However provision of meals alone may not ensure that nutritional needs are met. In one study two thirds of people in receipt of meals at home divided the meals provided for use on more than one occasion, suggesting continued overall insufficient food intake [31].

Nutritionally complete supplements are often a first line intervention and have been shown to have a positive effect on nutritional status [32] but mixed effects on body weight. In one study provision of an additional 600 kcal per day by supplements over a 12 week period resulted in a mean weight gain of +3.5 kg in intervention subjects (*p* < 0.001) [33]. The review by Potter and colleagues, quantified effects on body weight as 2.05% (95% CI 1.63 to 2.49) [32]. In contrast other studies report significant increases in energy and food intake but no significant weight gain [34,35,36,37,38,39]. However the efficacy of supplements is limited by taste intolerance which precludes their long-term use [34]. Evaluation of their efficacy in community settings in limited [40].

Food enrichment, defined as increasing the energy density of meals by adding energy rich foods, is an alternative to supplements and may suit older people, who often have small appetites. This approach may be more economical, avoid taste fatigue and allow continuation, and enjoyment, of usual eating patterns. Results from studies using this approach are however mixed. Two trials, one in hospital inpatients [41], the other in the community in a nursing home [42] increased energy provision by 200 kcal per day for 8 and 15 weeks respectively. In both trials energy intake increased significantly however no significant weight gain was observed in either study. Weight maintenance was achieved in the nursing home subjects receiving the intervention [42].

Another study in free-living adults with chronic obstructive pulmonary disease whose BMI was <20.0 kg/m^2^ or had a recent weight loss of >10%, looked at the effects of tailored dietary advice to increase energy intake along with the addition of milk powder. At 6 and 12 months body weight had increased significantly (+2.0 kg, SD 4.6; and +3.0 kg, SD 6.2 respectively) and positive improvements in quality of life and activities of daily living were observed [43]. Weight gain of (+1.3 (0.53) kg, *p* = 0.03) was observed in undernourished older people in a residential care home whose food was enriched, in comparison to weight loss in residents who continued with usual meals (−0.2 (1.5) kg, *p* = 0.54), between group difference were not significant, however the within group improvement in body weight suggests a positive effect from food enrichment [44].

Improvements in nutritional status and body weight were seen in free living older adults who were at risk of undernutrition after hospital admission, following 12 weeks of dietary enrichment [45].

Of course not all older people are undernourished and the prevalence of obesity in older people is rising. Anxieties exist regarding weight loss in the older adult. This arises from epidemiological evidence which suggests an association between lower BMI in older people and increased mortality [25,46]. However this is thought likely to be the result of unintentional weight loss as a consequence of conditions such as cancers, chronic heart and lung disease.

**Figure 2:**
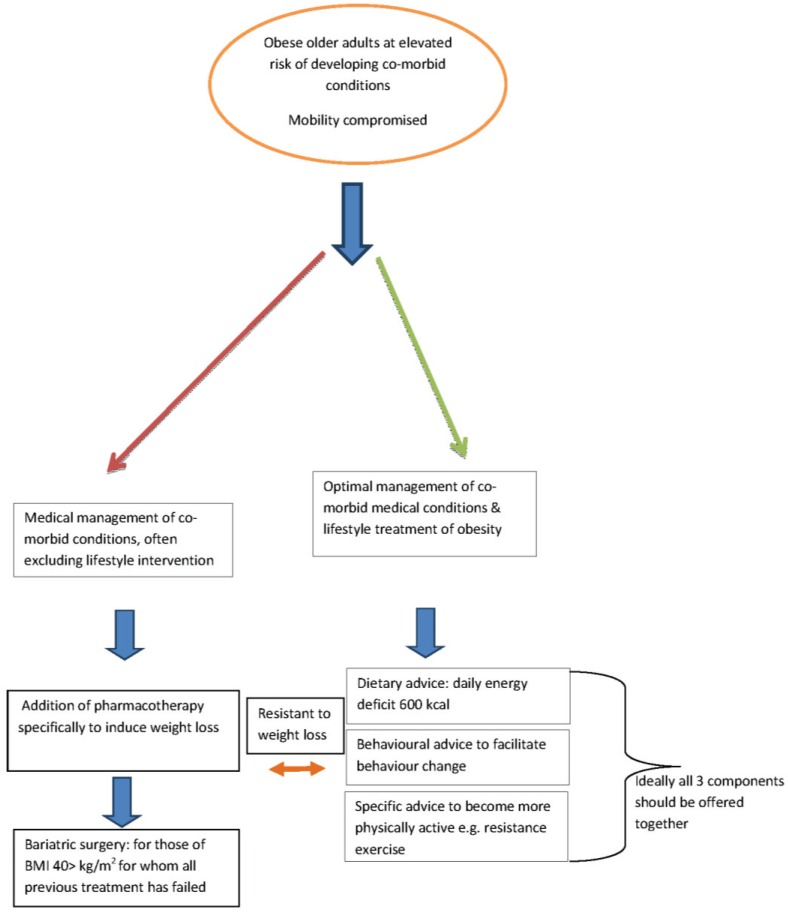
Schematic treatment strategy for obese older adults. Adapted from [26].

As in younger adults weight management is appropriate in older people and has been shown to reduce disease risk and improve quality of life (Table 2). Life style interventions should be the first step and should aim to achieve modest weight loss of 5%–10% (5–10 kg) using a balanced diet with a moderate daily energy deficit of 500–600 kcal daily. Given that the aging process results in loss of muscle mass, it is essential that weight loss programmes do not induce further loss resulting in the development of “sarcopenic obesity” where the adult has lowered muscle mass, within a given BMI and impaired functional capacity [47] Preservation of muscle mass can be achieved by the inclusion of an exercise/physical activity component in any weight management programme. Life style intervention studies in overweight and obese older adults which included both weight loss and physical activity report improvements in body composition (reduced fat mass and increased total lean mass), metabolic risk factors, functional status, well-being and a reduced degree of frailty [26]. A schematic treatment strategy is shown in Figure 2.

The inclusion of a weight maintenance component in weight management programmes is advocated by all clinical guidelines for weight management. However, it is suggested that weight maintenance in older adults following intentional weight loss may not always be required. A study in which obese and overweight adults were allocated to either a low intensity dietary counseling maintenance period, to exercise advice or to usual care/control found no differences in body weight changes between groups. The lack of difference between the groups suggests that older adults who were sufficiently motivated to achieve intentional weight loss were able to commit to weight maintenance without formal direction [48]. A systematic review [49] which examined the effectiveness of weight management approaches in older adults found the effect of lifestyle advice and guidance in the older obese or overweight adult on body weight was maintained at around ~2 kg at both one and two years follow-up.

**Table 2:** Potential benefits and risks related to intentional weight loss in the older adults Adapted from [50].

Potential benefits	Potential risks
Adults with impaired glucose tolerance less likely to become diabetic	
Improved cardiovascular risk factors Reduced use of chronic medications if 10% weight loss is achieved	
Improved respiratory health Decrease in sleep apnoea	Compromised micronutrient status resulting from poor diet quality
Activities of daily living improved or remain constant Maintain or improve activities of daily living	Loss of lean muscle tissue (sarcopenia) which can be challenged by undertaking regular physical activity
Improved quality of life	Gallstone formation in a minority of adults as a result of profound weight loss (>20 kg)

## 8. Conclusions

While many older adults remain healthy and eat well, those in poorer health may experience difficulties in meeting their nutritional needs. Meeting the diet and nutrition needs of older people is crucial for the maintenance of health, functional independence and quality of life.

In the UK, future health policy is aimed at shifting the balance of care towards the community and it is essential that nutritional needs of older adults are explored and addressed. Failure to do this is likely to lead to a loss of independence with subsequent increased demands on social care provision and increased hospital admissions with the potential for more invasive and expensive healthcare requirements.
